# Case report: The clinical utility of metagenomic next-generation sequencing in mucormycosis diagnosis caused by fatal *Lichtheimia ramosa* infection in pediatric neuroblastoma

**DOI:** 10.3389/fped.2023.1130775

**Published:** 2023-06-19

**Authors:** Huili Shen, Xiaodi Cai, Jing Liu, Gangfeng Yan, Ying Ye, Rui Dong, Jufang Wu, Li Li, Quanli Shen, Yutong Ma, Qiuxiang Ou, Meili Shen, Weiming Chen, Guoping Lu

**Affiliations:** ^1^ Pediatric Intensive Care Unit, Children’s Hospital of Fudan University, National Center for Children’s Health, Shanghai, China; ^2^ Dermatological Department, Children’s Hospital of Fudan University, National Center for Children’s Health, Shanghai, China; ^3^ Surgical Oncology Department, Children’s Hospital of Fudan University, National Center for Children’s Health, Shanghai, China; ^4^ Institute of Antibiotics, Huashan Hospital, Fudan University, Shanghai, China; ^5^ Lab. of Mycology, Department of Dermatology, Huashan Hospital, Fudan University, Shanghai, China; ^6^ Radiology Department, Children’s Hospital of Fudan University, National Center for Children’s Health, Shanghai, China; ^7^ Medical Department, Nanjing Dinfectome Technology Inc., Nanjing, China

**Keywords:** *Lichtheimia ramosa*, mucormycosis, pediatric, neuroblastoma, mNGS (metagenomic next-generation sequencing)

## Abstract

*Lichtheimia ramosa* (*L. ramosa*) is an opportunistic fungal pathogen of the order *Mucorales* that may result in a rare but serious mucormycosis infection. Mucormycosis could be angioinvasive, causing thrombosis and necrosis in the nose, brain, digestive tract, and respiratory tract. The infection is highly lethal, especially in immunocompromised hosts, and the incidence has been on the rise. However, due to its relatively low incidence in pediatric population and the challenges with diagnosis, the awareness and management experience for pediatric mucormycosis are extremely limited, which might lead to poor outcomes. In this study, we comprehensively reviewed the course of a fatal rhinocerebral mucormycosis case in a pediatric neuroblastoma patient receiving chemotherapy. Due to a lack of awareness of the infection, the standard care of amphotericin B treatment was delayed and not administered until the identification of *L. ramosa* by metagenomic next-generation sequencing (mNGS)-based pan-pathogen detection of the patient's peripheral blood sample. We also reviewed the literature on *L. ramosa* infection cases reported worldwide between 2010 and 2022, with an analysis of clinical manifestation, prognosis, and epidemiological data. Our study not only highlighted the clinical value of comprehensive mNGS in rapid pathogen detection but also raised awareness of recognizing lethal fungal infection early in immunocompromised hosts including pediatric cancer patients.

## Introduction

Mucormycosis is a highly lethal infection caused by the opportunistic fungal pathogens of the *Mucorales* order, usually in patients with malignancies, transplantation, and diabetes (1). It is the world's third most common invasive mycosis after candidiasis and aspergillosis (2). The clinical management of mucormycosis has drawn increasing attention, accompanying the growing number of invasive mucormycosis reported during the present coronavirus disease (COVID-19) pandemic (3). Based on the infection manifestations, it can be classified into rhinocerebral, pulmonary, gastrointestinal, cutaneous, disseminated, and uncommon types (1). Rhinocerebral mucormycosis refers to mucormycosis that enters the nasal cavity through the junction of skin and mucosa, then spreads to the palate, sinus, and orbit, and finally causes intracranial infection due to vascular invasion or bone destruction. The most common pathogens of mucormycosis are *Rhizopus spp.*, *Mucor spp.*, and *Lichtheimia spp.* (4). *Rhizopus spp.* is currently the most common pathogen of mucormycosis worldwide, especially in rhinocerebral mucormycosis.

The nomenclature and taxonomy of subphylum *Mucoromycotina* have been evolving in recent years. In the subphylum of *Mucoromycotina*, *Absidia corymbifera* was a common clinical pathogen, especially in the secondary infection of skin lesions after severe trauma or burn. In 2007, Hoffman et al. (5) classified thermotolerant *Absidia* as *Mycocladus*, which then comprises the three species of *M. corymbifer*, *M. blakesleeanus*, and *M. hyalospora* based on their morphological, physiological, and phylogenetic characteristics. In 2009, the genus *Mycocladus* was renamed *Lichtheimia* and divided into two species, *L. corymbifera* and *L. ramosa*, and the former was found to be more resistant to amphotericin B (6). In 2010, the genus *Lichtheimia* was further classified into five species: *L. corymbifera*, *L. ornata*, *L. ramosa*, *L. hyalospora*, and *L. sphaerocystis*, by their molecular markers, mating tests, morphology, and growth rate (7). The first three species have been reported to be clinically relevant.

The incidence of mucormycosis has been studied globally showing a general trend of increase and geographic differences (8–10). Though lacking statistics on the exact burden of mucormycosis, the prevalence has been estimated based on population or hospital-based studies worldwide and the differences between developed and developing countries were observed, resulted from multiple factors including risk groups, healthcare accessibility, clinical management and intervention (11). Generally speaking, the incidence of mucormycosis in pediatric population is relatively lower than that in adults, which lead to less awareness of the disease regarding the clinical diagnosis and limited experience in disease management (12). Similar to adult patients, the common risk factors in children include hematologic malignancy, stem cell/solid organ transplant, autoimmune diabetes mellitus, premature infants, and so on (13). However, diagnosing pediatric mucormycosis is very challenging due to the lack of straightforward testing approaches and complicated clinical manifestations and symptoms (14).

At present, the treatment mainly relies on amphotericin B. However, the renal and hepatic toxicity of amphotericin B formulations should be taken into account, especially for immunodeficient patients, and therefore, a modified dose should be considered in intolerant cases (15, 16). Early diagnosis, timely treatment with prescribed drugs, and surgical operations are therefore essential. In this study, we reported the disease course of a lethal *L.ramosa* infection in a pediatric cancer patient receiving chemotherapy. The empirical diagnosis and treatment were initially suboptimal due to a lack of awareness of the infection until the identification of *L.ramosa* by metagenomic next-generation sequencing (mNGS). The patient died two days later due to multiple organ failures. We also performed the first comprehensive literature review of *L. ramosa* infection cases documented between 2010 and 2022 to augment the understanding of the clinical manifestation, prognosis, and risk factors of mucormycosis.

## Methods

To comprehensively study the world-wide reported mucormycosis cases, we searched for literatures in PubMed, Ovid MEDLINE, Embase, WANFANG, and CNKI Database. Due to the redefinition and classification of *Lichtheimia* in 2010 (7), we decided to focus on the publications between January 2010 and February 2022 with any of the following key words: *Lichtheimia ramosa, ramosa, Lichtheimia,* mucormycosis, *Absidia corymbifera, Mycocladus corymbifera, L. corymbifera, L. ornata, L. ramosa, Absidia ramosa, Rhizopus ramosus, Mucor ramosus, L. hyalospora, L. blakesleeaana,* and *L. sphaerocystis.* Only the cases with confirmed *L. ramosa* infection were included, whose clinical and epidemiological data were summarized in Table 1.

**Table 1:** Summary of *L.ramosa* infection cases reported between 2010 and 2022.

Case	Year	Age/Sex	Geography	Underlying disease	Site of infection	Clinical manifestation	Method of identification	Treatment	Outcome
1 (17)	2010	10/F	Spain	2nd relapse of AML	Pulmonary	Cough, tough respiratory, agranulocytosis	Smear staining, histopathology, sequencing	Unspecified	Death
2 (18)	2013	32/M	Greece	Automobile accident with large deep wound	Cutaneous	A subcutaneous cavity in the left lumbar region with abscess	Microscopy, histopathology, PCR sequencing	Surgical debridement, antimycotic solution cleansing, broad-spectrum antibiotics	Cured
3 (19)	2013	84/M	France	Severe arterial occlusion of left lower limb with severe sepsis, type 2 diabetes	Cutaneous	Gray—white deposits on amputation wounds	Microscopy, histopathology, gene sequencing	Surgical debridement, LAMB, calcium alginate	Cured
4 (20)	2013	27/M	France	Acute leukemia, hand injury and fasciitis	Cutaneous	Fasciitis	Pathology, histopathology	LAMB	Death
5 (21)	2013^1^	21/M	France	Automobile accident with multiple compound fractures	Cutaneous	Green, mouldy discharges	Histopathology	LAMB, partial amputation	Cured
6 (21)	2013^1^	72/M	France	Bilateral amputation for acute ischemia	Cutaneous	Purulent, mouldy discharges	Histopathology, microscopy	LAMB	Death
7 (22)	2013	59/M	France	Non-Hodgkin lymphoma, diabetes mellitus	Kidney	Unspecified	Histopathology, qPCR	LAMB	Cured
8 (23)	2013	52/M	Turkey	AIDS with antiretroviral therapy	Pulmonary	Systemic symptoms mainly at lung	Histopathology, PCR	Amphotericin B deoxycholate, LAMB	Death
9 (24)	2013	4/F	France	Chronic intestinal obstruction syndrome, multiple organ transplantation	Transplanted stomach	Digestive tract perforation, massive gastric bleeding	Pathology, PCR	LAMB	Cured
10 (25)	2013–2016	50/F	France	Burn (60%)	Cutaneous	Unspecified	Histopathology, qPCR	LAMB, caspofungin	Death
11 (25)	2013–2016	63/F	France	Burn (35%)	Cutaneous	Unspecified	Histopathology, qPCR	No drug used	Cured
12 (25)	2013–2016	42/M	France	Burn (50%)	Cutaneous	Unspecified	Histopathology, qPCR	LAMB, caspofungin	Cured
13 (25)	2013–2016	65/F	France	Burn (40%)	Cutaneous	Unspecified	Histopathology, qPCR	No drug used	Cured
14 (25)	2013–2016	43/M	France	Burn (80%)	Cutaneous	Unspecified	Histopathology, qPCR	LAMB, caspofungin	Cured
15 (26)	2014	20/F	India	Burn (60%)	Cutaneous	Empyrosis, shock	Biopsy, histopathology	Surgical debridement, amphotericin B deoxycholate, Imipenem	Cured
16 (27)	2014^2^	n.a.	India	Unspecified	1 bronchoalveolar lavage fluid, 1 pulmonary, 1 cutaneous	Unspecified	DNA sequencing	Unspecified	Unspecified
17 (28)	2014	55/M	Egypt	Solid organ transplant	Pulmonary	Unspecified	Histopathology, microscopy DNA sequencing	LAMB	Death
18 (28)	2014	45/M	Egypt	Diabetes	Pulmonary	Unspecified	Histopathology, microscopy DNA sequencing	LAMB, itraconazole	Cured
19 (28)	2014	55/M	Egypt	Diabetes	Pulmonary	Unspecified	Histopathology, microscopy DNA sequencing	LAMB, itraconazole	Cured
20 (29)	2015^3^	n.a.	India	Unspecified, may have empryrosis and diabetes	3 Rhinocerebral, 1 Cutaneous	Unspecified	Microscopy, histopathology	LAMB or Amphotericin B deoxycholate	1 death 3 cured
21 (30)	2015	8/M	Japan	AML relapse, chemotherapy, hematopoietic stem cell transplantation	Disseminated	Pneumonia, diffuse thickening of alveolar walls with various inflammatory cells	Histopathology, sequencing	LAMB, cord blood transplantation	Death
22 (31)	2016	56/M	Spain	Diabetes, H1N1 infection	Disseminated	Bronchitis, pulmonary thromboembolism	Post-mortem examination	meropenem, levofloxacin, oseltamivir, cotrimoxazole, linezolid, voriconazole, corticosteroids	Death
23 (32)	2016	7/M	USA	Chronic granuloma, post-transplantation, immunosuppression	Pulmonary	Vascular embolism	Microscopy	LAMB	Death
24 (32)	2016	5/M	USA	Chronic granuloma, pulmonary nodules	Pulmonary	Vascular embolism	Microscopy	LAMB	Cured
25 (33)	2017	41/M	Spain	Acute myeloid leukemia, chemotherapy, neutropenia	Pulmonary	Unspecified	Histopathology, pathology, microscopy	LAMB, Posaconazole, surgery	Cured
26 (33)	2017	61/M	Spain	Lymphoblastic acute leukemia, chemotherapy, neutropenia, trauma	Cutaneous	Unspecified	Histopathology, pathology, microscopy	LAMB, Posaconazole, surgery	Cured
27 (33)	2017	72/F	Spain	Surgery, squamous cell carcinoma	Cutaneous	Unspecified	Histopathology, pathology, microscopy	LAMB, surgery	Cured
28 (33)	2017	49/M	Spain	Diffuse large B-cell lymphoma, haploidentical HSCT, neutropenia	Disseminated	Unspecified	Histopathology, PCR, microscopy	LAMB, Posaconazole, micafungin	Remission
29 (34)	2018	53/F	Japan	Esophageal cancer, pneumococcal pneumonia, transverse colon perforation	Colon	Unspecified	PCR	LAMB	Cured
30 (35)	2018	43/M	China	Surgery on nose bridge eight years ago	Cutaneous	Chronic granulomatous disease on face	Histopathology	Amphotericin B (unspecified)	Cured
31 (36)	2020	38/M	Netherlands	Kidney transplantation	Transplanted kidney	Kidney pain	Pathology	LAMB, antifungal induction therapy, debridement, lavage	Cured
32 (37)	2020	n.a.	USA	COVID-19	Unspecified	Unspecified	cfDNA	Unspecified	Unspecified
33 (38)	2021	53/M	China	Tooth extraction	Rhinocerebral	Fever, headache, slurred speech, brainstem failure	CFS mNGS	LAMB	Remission
34 (39)	2021	5/M	Argentina	Seizures and liver failure, medication of valproic acid,	Cutaneous	Round necrotic lesions with black margins in chin area, posterior involvement of soft tissue	Pathology, PCR, histopathology	Debridement, LAMB	Cured
35 (40)	2021	Late 50s/M	Netherlands	COVID-19	Pulmonary	Pulmonary cavities and a reversed halo sign by CT	Histopathology	LAMB, posaconazole	Death
36 (41)	2021	46/M	Spain	COVID-19, kidney transplantation, arterial hypertension	Musculoskeletal	Pain, hematoma of lower right limb, muscle tissue necrosis	Histopathology	LAMB, isavuconazol, debridement	Cured

LAMB, liposomal amphotericin B; AML, acute myeloid leukemia; CSF, cerebrospinal fluid; mNGS, metagenomic next-generation sequencing; CT, computed tomography; n.a/, not available; cfDNA, cell-free DNA.

^1^ Possible person-to-person transmission in intensive care.

^2^ 3 of 54 cases were detected with *L. ramosa*.

^3^ 4 of 38 cases in this study infected with *L. ramosa*, individual details were not specified.

## Results

### Case presentation

**Figure 1:**
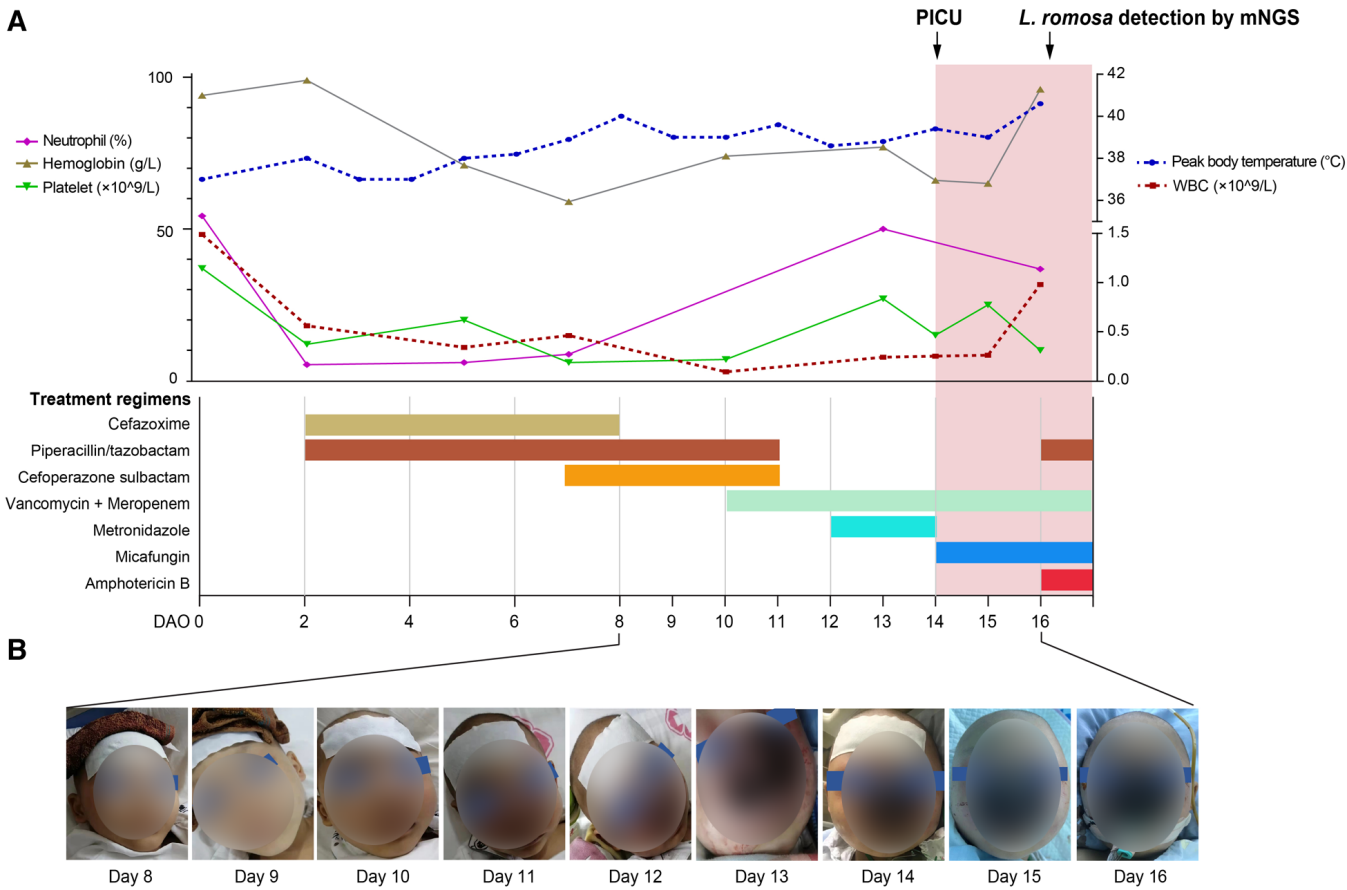
Timeline of the patient's disease course. (**A**) The patient's treatment course and clinical testing data. Routine blood workup results were shown in the top panel, including neutrophil, hemoglobin, and platelet on the left and white blood cells (WBCs) on the right. The patient's treatment course was shown in the bottom panel. (**B**) Images showing the development of the rhinocerebral *L. ramosa* infection from DAO 8 to DAO 16.

A 6-year-old male patient developed a fever accompanied by nasal bleeding and a small amount of vomiting. He was admitted to a local hospital on October 6, 2021 (Day 0 after symptom onset, DAO 0). Before this hospital admission, the patient was admitted to the hospital in July 2019 due to a six-month poor appetite and significant weight loss, as well as right leg pain for 20 days. He was then diagnosed of neuroblastoma accompanied by intracranial and multiple bone metastases after a complete bone marrow biopsy. The patient completed three cycles of maintenance chemotherapy for high-risk neuroblastoma and radical resection of the retroperitoneal tumor under general anesthesia. Starting from DAO 0, routine peripheral blood testings were performed regularly to monitor the patient's conditions, which showed constantly low levels of white blood cells, neutrophils, platelets, and Hemoglobin (Figure 1A). Up till DAO13, the patient was empirically suspected of having a bacterial infection post-chemotherapy and treated as follows (Figure 1A): piperacillin/tazobactam (45 mg/kg, q8h), cefazoxime (50 mg/kg, bid), cefoperazone sulbactam (50 mg/kg, q12h), meropenem (20 mg/kg, q8h), vancomycin (10 mg/kg, q6h), and metronidazole (7.5 mg/kg, q8h) treatment. The patient also received the infusion of platelet (1 U) on DAOs of 3, 7, and 12, hemoglobin (1.5 U) on DAOs of 8 and 11, and recombinant human granulocyte colony-stimulating factor (rhGCSF, 150 µg, qd) on DAOs of 2–13. After the whole antibacterial treatment, the patient still experienced intermittent fever, nasal bleeding, and mouth pain (Figures 1A,B). The C-Reactive protein (CRP) level increased to 103.5 mg/L (DAO 10) and 181.0 mg/L (DAO 13). The conventional culture with blood samples at the local hospital failed to detect the presence of any possible causative pathogens.

**Figure 2:**
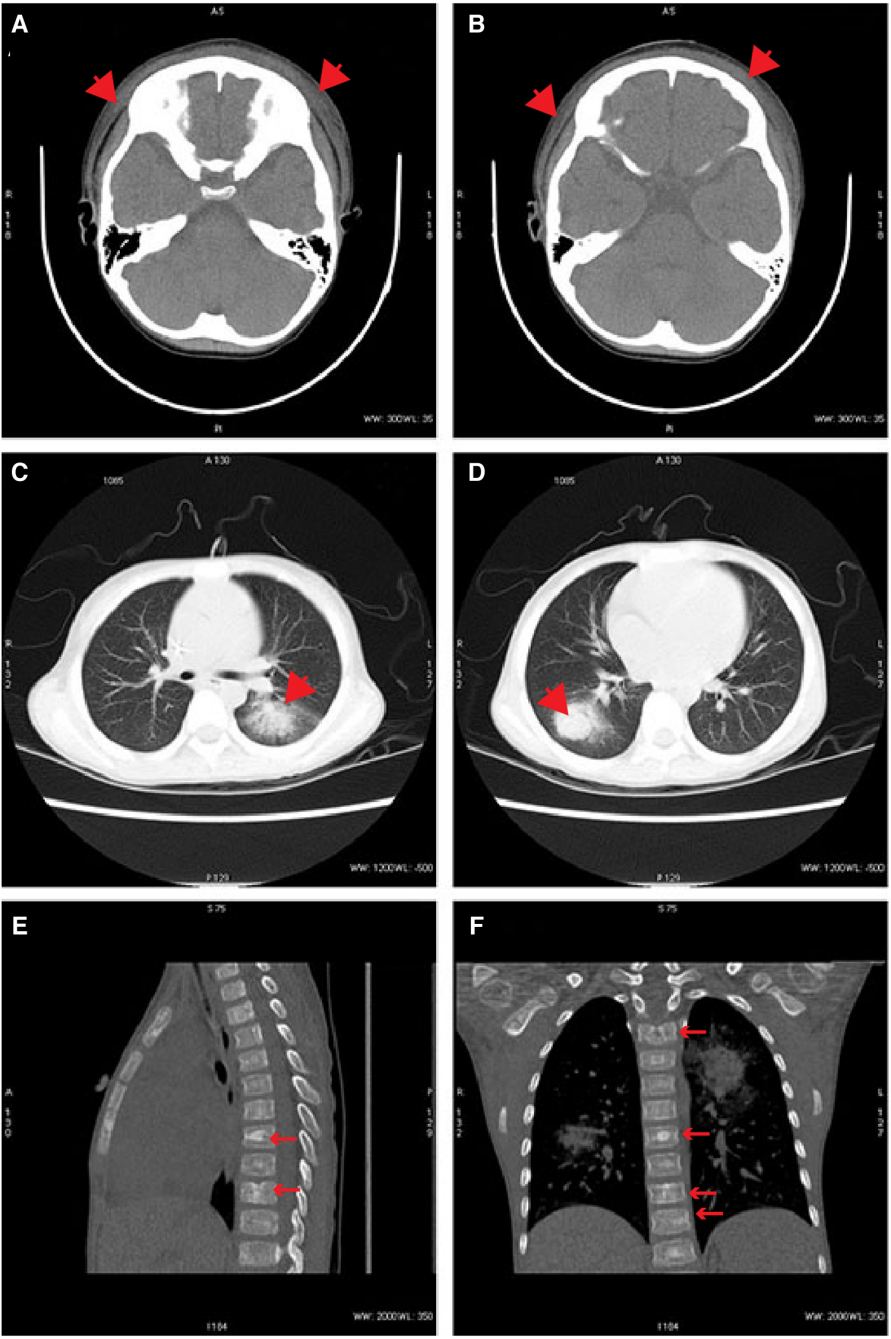
Representative radiographs. (**A,B**) CT images demonstrating scalp abscess on DAO 14. (**C,D**) CT images demonstrating pulmonary infarctions on DAO 14. (**E,F**) CT images demonstrating evidence of osteomyelitis on DAO 14.

**Figure 3:**
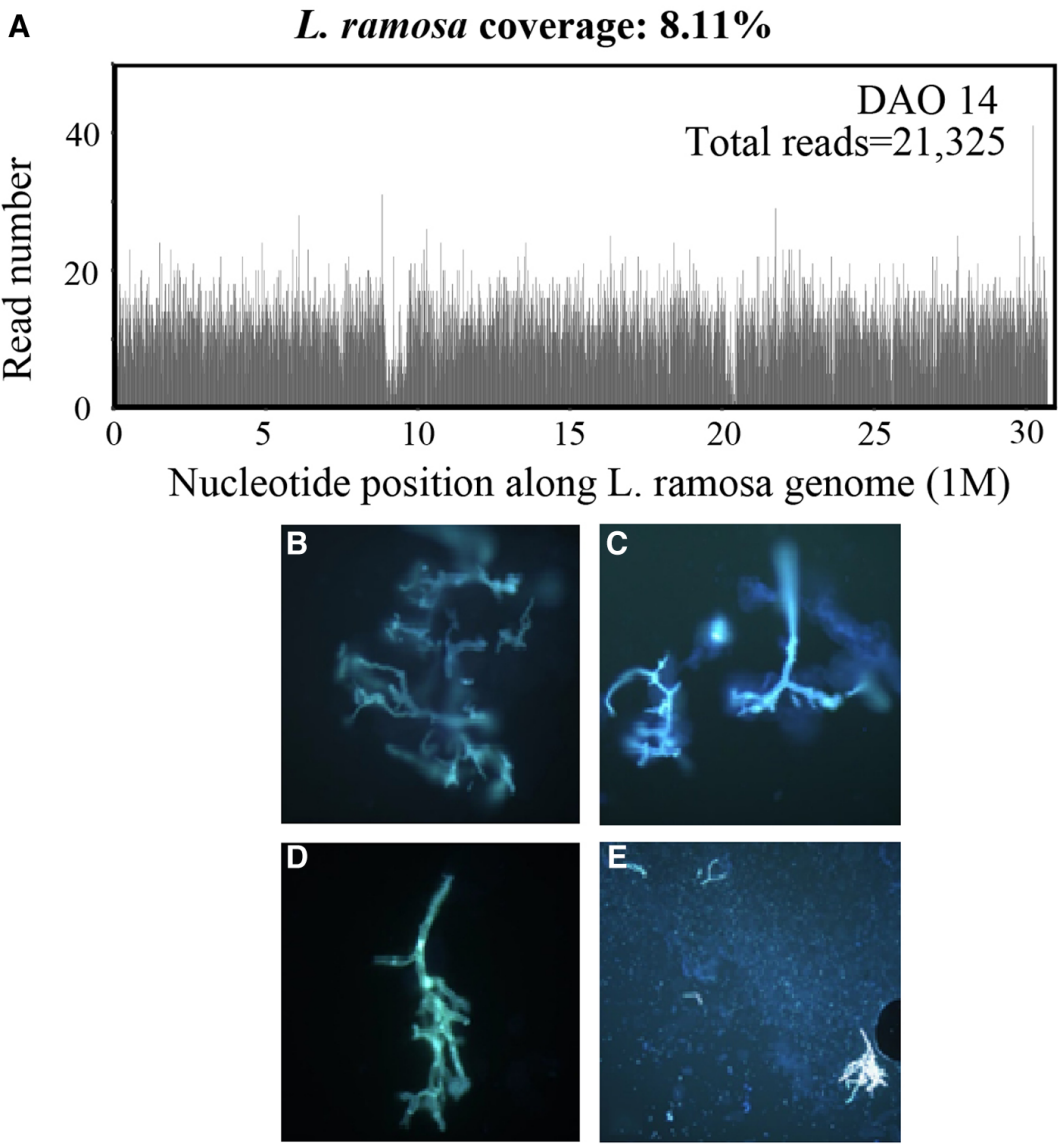
Laboratory investigations. (**A**) mNGS results showing the read depth throughout the *L. ramosa* genome derived from the patient's blood sample on DAO 14. (**B–E**) Fluorescence staining of the tissue samples collected from the nasal gangrene region. Microscopic examination showed direct evidence of presence ribbon-shaped broad sparsely septate hyphae (**B–D**, 400×) and clusters of sporangiospores (**E**, 200×), suggestive of the *Mucorales* order.

On DAO 14, the patient was admitted to Pediatric Intensive Care Unit (PICU), Children's Hospital of Fudan University, due to severe conditions, including a high fever up to 39.2°C, a small amount of blood oozing from both eyes, and progressive gangrene. The standard cultivate experiments were performed with blood, throat swab, and anal swab samples, but all returned negative results. (1–3)-β-D-Glucan (G) and glactomannan (GM) tests and lipopolysaccharides (LPS) were within the normal range but the evaluated levels of procalcitonin (PCT, 4.27 ng/ml) and Interleukin 6 (IL-6, 3.57 ng/ml) were reported. Computed tomography (CT) showed scalp abscess (Figures 2A,B), pulmonary infarction (Figures 2C,D), and evidence of osteomyelitis (Figures 2E,F). The following treatments were given to relieve the severe symptoms (Figure 1A): meropenem (40 mg/kg, q8h, three days), vancomycin (15 mg/kg, q6h, three days), piperacillin/tazobactam (112.5 mg/kg, q8h, one day), micafungin (2 mg/kg, qd, three days), infusion of platelet (1 U, DAO 14), hemoglobin (1 U, DAO 15), and albumin (10 g, DAOs 14–16), and rhGCSF (150 µg, qd, DAOs 2–13). However, the patient showed no noticeable improvement (Figures 1A,B), and his CRP remained above 160 mg/l. In the meantime, the peripheral blood sample was subject to metagenome next-generation sequencing (mNGS) to comprehensively search for possible pathogens (DAO 14). On DAO 15, the patient received rescue, endotracheal intubation, and vasoactive drug therapy due to worsened conditions. On DAO 16, *L. ramosa* was identified by mNGS with unique DNA sequences covering 8.11% of the *L. ramosa* genome (Figure 3A), supporting the diagnosis of mucormycosis by *L. ramosa* infection. Amphotericin B deoxycholate (5 mg/kg, ivgtt) was immediately administered, and tissue samples were collected from the nasal gangrene region for microscopic examination, which confirmed the diagnosis of *L. ramosa* infection shortly thereafter (Figures 3B–E). Despite the immediate treatment by amphotericin B after the detection of *L. ramosa*, the patient died of severe multiple organ failure on DAO 16.

### Literature review

We retrieved 25 publications with a total of 41 L*. ramosa* infectious cases (Table 1) where the majority were from Europe (22/41, 53.7%), followed by 12 from Asia (29.3%), 4 from America (9.8%), and 3 from Africa (7.3%). The most common sites of infection were cutaneous (*N* = 17) and pulmonary (*N* = 10). As expected, most cases were immunocompromised subjects with underlying risk factors such as cancer (9, 22.0%), diabetes (6, 14.6%), burn (6, 14.6%), and organ transplantation (5, 12.2%). Excluding eight cases with unknown age at diagnosis, the median age was 46 years old (range: 4–84) and six of them were under 10 years old. More specifically, five of the six pediatric cases were treated with liposomal amphotericin B (LAMB) and three were cured. While among all treatment-specified cases, 32 of them received the Amphotericin B treatment which successfully cured 72% (23/32) of the patients. Meanwhile, we observed a trend of adopting NGS-based methods in pathogen detection and more recently, a rising incidence in COVID-19 patients worldwide after 2020.

## Discussion

In the past decades (42), mucormycosis incidence has increased rapidly, with the overall mortality rate as high as 90%. Mucormycosis mainly occurs in immunocompromised patients with conditions such as diabetes mellitus, malignancies, burns, autoimmune diseases, penetrating trauma or receiving corticosteroids etc (1). Notably, several studies have witnessed a resurgence of mucormycosis during the present global COVID-19 pandemic, which indicated that the accompanied immunocompromised state and steroid use in COVID-19 patients were the risk factors underlying mucormycosis (43, 44).

The prevalence of diagnosed infection shows a geographical difference, mainly distributed in Europe (68.2%), followed by Asia (16%) and Africa (9%) (45, 46). In developed countries, the number of *Lichtheimia* infection cases is high among hematopoietic stem cell transplantation recipients and hematological malignancies. In contrast, mucormycosis is more associated with diabetes mellitus and ketoacidosis in developing countries (46).

To our best knowledge, we reported the first case of mucormycosis by *L. ramosa* in a pediatric neuroblastoma patient, who had received chemotherapy prior to *L. ramosa* infection and neuroblastoma is the most common extra-cranial solid tumor in infants and children and represents 8%–10% of all childhood tumors (47). Based on our literature review, cancer is an important risk factor underlying *L. ramosa* infection in both pediatric and adult populations. A total of six pediatric *L. ramosa* infection cases were included in our literature review and both of the AML relapsed children died after infection (17, 30). In addition, early diagnosis and treatment are essential for the cure, as demonstrated by the cases collected in our study. For instance, Cases 23 and 24 are brothers who were both infected by *L. ramosa* (32). The older patient died, while the younger patient received early detection and timely treatment, leading to a favorable prognosis.

For now, the gold standard for diagnosis is histopathology testing, mainly with tissue samples. Traditional invasive procedures for etiologic diagnosis have limitations such as patient instability (37). In addition, the positive rate of histopathological and microbiological methods from cultivated clinical samples, is around 50% or even lower due to the morphological similarity between different *Lichtheimia* species (29–48). Therefore, molecular testing such as polymerase chain reaction (PCR) or NGS-based technologies using serum samples are essential supplemental tools for diagnosis. In our case, the local hospital lacked awareness of rare fungal infections and access to molecular tests such as mNGS, leading to the delay of diagnosis and treatment. The presented case also indicated the lethality of *L. ramosa* infection in pediatric cancer patients, and we believe a more timely etiologic diagnosis when the initial pan-antibiotics application was ineffective could result in a favorable outcome. Thus, mNGS is highly recommended for diagnostically challenging cases with undetermined infection and complex manifestations, especially for pediatric patients, which enables rapid and affordable pan-pathogen screening to guide targeted intervention against deadly infections. Admittedly, mNGS has limitations, especially the false-positive results caused by contamination. Also, the reference database selection and interpretation may affect the pathogen detection (49). Thus, alternative methods are needed to validate mNGS findings.

Amphotericin B is considered the first choice for mucormycosis (15). Indeed, 75.0% (24/32) of the patients that received the treatment of amphotericin B recovered from *L. ramosa* infection in our literature review. Furthermore, combining amphotericin B and surgical debridement of infected tissues was reported to be able to improve the cure rate. For example, Schneidawind et al. (50) reported three acute myeloid leukemia (AML) patients complicated with pulmonary mucormycosis who were successfully treated by combined LAMB and surgical resection before stem cell transplantation (SCT). Similarly, LAMB treatment effectively prevented infection recurrence despite immunosuppressive drugs in an SCT case with pulmonary mucormycosis after surgical resection (51). Alternatively, posaconazole has been reported as a salvage treatment for amphotericin B refractory patients, but it is less effective and more likely to cause resistance (52).

In conclusion, we reported a rhinocerebral mucormycosis pediatric case caused by *L. ramosa* with neuroblastoma and reviewed the *L. ramosa* infection cases published between 2010 and 2022. The limited number of clinical cases, lack of awareness, and technical challenges of detecting different infection sites may restrict the interpretation of the literature analysis. Our case demonstrates the importance of rapid pan-pathogen screening by mNGS to guide timely treatment selection against fast-developing, fatal infections for pediatric cancer patients.

## Data Availability

The datasets presented in this article are not readily available because of ethical and privacy restrictions. Requests to access the datasets should be directed to the corresponding authors.
